# Construction of a questionnaire measuring outpatients' opinion of quality of hospital consultation departments

**DOI:** 10.1186/1477-7525-2-43

**Published:** 2004-08-04

**Authors:** Isabelle Gasquet, Sylvie Villeminot, Carla Estaquio, Pierre Durieux, Philippe Ravaud, Bruno Falissard

**Affiliations:** 1 Delegation of innovation, health security and assessment, Direction of medical policy – Assistance Publique-Hôpitaux de Paris, 3 avenue Victoria 75184 Paris cedex, France; 2 UPRES JE 2360 (PSIGIM : Paris South Innovation Group in Mental health), Paul Brousse hospital, Villejuif, France; 3 Department of Public Health and Medical Informatics, Faculté de Médecine Broussais Hôtel Dieu, Paris, France; 4 Department of Epidemiology, Biostatistics et and clinical research, Hôpital Bichat Assistance Publique-Hôpitaux de Paris, Paris, France

**Keywords:** Patient satisfaction, quality of care, hospital, consultation, psychometrics

## Abstract

**Background:**

Few questionnaires on outpatients' satisfaction with hospital exist. All have been constructed without giving enough room for the patient's point of view in the validation procedure. The main objective was to develop, according to psychometric standards, a self-administered generic outpatient questionnaire exploring opinion on quality of hospital care.

**Method:**

First, a qualitative phase was conducted to generate items and identify domains using critical analysis incident technique and literature review. A list of easily comprehensible non-redundant items was defined using Delphi technique and a pilot study on outpatients. This phase involved outpatients, patient association representatives and experts. The second step was a quantitative validation phase comprised a multicenter study in 3 hospitals, 10 departments and 1007 outpatients. It was designed to select items, identify dimensions, measure reliability, internal and concurrent validity. Patients were randomized according to the place of questionnaire completion (hospital v. home) (participation rate = 65%). Third, a mail-back study on 2 departments and 248 outpatients was conducted to replicate the validation (participation rate = 57%).

**Results:**

A 27-item questionnaire comprising 4 subscales (appointment making, reception facilities, waiting time and consultation with the doctor). The factorial structure was satisfactory (loading >0.50 on each subscale for all items, except one item). Interscale correlations ranged from 0.42 to 0.59, Cronbach α coefficients ranged from 0.79 to 0.94. All Item-scale correlations were higher than 0.40. Test-retest intraclass coefficients ranged from 0.69 to 0.85. A unidimensional 9-item version was produced by selection of one third of the items within each subscale with the strongest loading on the principal component and the best item-scale correlation corrected for overlap. Factors related to satisfaction level independent from departments were age, previous consultations in the department and satisfaction with life. Completion at hospital immediately after consultation led to an overestimation of satisfaction. No satisfaction score differences existed between spontaneous respondents and patients responding after reminder(s).

**Conclusion:**

Good estimation of patient opinion on hospital consultation performance was obtained with these questionnaires. When comparing performances between departments or the same department over time scores need to be adjusted on 3 variables that influence satisfaction independently from department. Completion of the questionnaire at home is preferable to completion in the consultation facility and reminders are not necessary to produce non-biased data.

## Background

Medical care aims not only to improve health status but also to respond to patient needs and wishes and to ensure their satisfaction with care [1]. Likewise, conducting surveys to measure satisfaction with psychometrically validated questionnaires entails assessment of the quality of care organization and procedures [2]. Patient judgement on medical care also contributes to medical outcome. In the case of ambulatory care, it has been clearly shown that satisfied patients are more likely to cooperate with treatment, to maintain a continuing relationship with a practitioner [3] and thus enjoy a better medical prognosis [4].

From a conceptual point of view, the construct of patient satisfaction as been defined by Ware as an "attempt to capture the personal evaluation of care that cannot be known by observing care directly" and to consider opinion of patients as a multidimensional subjective indicator of quality of care [5]. The model most commonly, though implicitly, used in satisfaction work is the discrepancy model (degree of fulfillment of expectation is related to satisfaction level) giving to patient expectations a central role [6]. This model, according to Sitzia " implies that concentrating upon areas of expressed dissatisfaction is more valuable than obtaining consistency of expressed satisfaction" [4,7].

In France, measuring satisfaction has been mandatory since 1996 and several questionnaires have been developed to evaluate inpatient care [8-12]. Most existing outpatient satisfaction questionnaires have been developed to assess primary care practice, especially general practice [13-20]. However, it could be hypothesized that content of questionnaires evaluating primary care physician may be different from that of questionnaires exploring hospital consultation with a specialist because of differences in patient expectations. So it could be assumed that dimensions that are very important in the case of primary care like human qualities of the physician and medical information could have a lesser importance in case of hospital consultation, while technical competency could have a more important place [21-23].

Few questionnaires have been developed for hospital consultations. Of these, some were specific to one type of consultation like oncology [24], rheumatology [25] or diabetes clinics [21], while others were non-generic questionnaires [14]. There is one French-language questionnaire on satisfaction with outpatient hospital care, however this questionnaire was developed from an "expert" viewpoint [26]. Hence the decision to construct a complementary "patient-oriented" questionnaire implicating potential respondents in the generation and selection of items. Even if health care organization differs across countries, the role of the hospital in most countries is very similar and it could be expected that the questionnaire developed in France could be used in other countries with a public health system, in particular European countries.

The main objective was to develop, according to psychometric standards, a generic outpatient satisfaction questionnaire that could be used to compare hospital outpatient departments one with another or the same department over time. The questionnaire needed to be brief, understandable and easy to complete for outpatients aged18 years or older in medicine, surgery and psychiatric hospital consultations. It was designed to be self-administered. The French final version is being adapted in English, German, Italian, Spanish and Hebrew.

The secondary objective was to define administration procedures in routine study that minimize non-response bias. Three situations were tested: i) questionnaire issued and completed at hospital, immediately after consultation; ii) questionnaire mailed and completed at home before any reminder; iii) questionnaire mailed and completed at home only after reminder(s). The groups were compared for satisfaction.

### Overview of the questionnaire development

It comprised 2 phases. First, a qualitative phase for item generation and construction of a first version of the questionnaire (41-item version). Secondly, a quantitative phase comprising 2 steps. A first validation phase that provided a shortened version of the questionnaire (27-item version). Second, a replication validation phase to corroborate results from the previous steps. Finally a very short-form version (9-item) was constructed. All versions are presented in the Appendix (see additional file 1).

A steering committee supervised the questionnaire development procedure, comprising methodologists, hospital practitioners and persons from patient associations defending health care user rights. All analyses were performed using SAS software (version 8).

## Method

### Qualitative phase of item generation

A psychologist conducted 25 individual semi-structured interviews with recent outpatients, using the critical analysis incident technique [27]. Subjects were asked to detail specific events they had experienced and situations associated with neutral, pleasant or unpleasant emotions that had influenced their opinion on consultation. An interview guide constructed according to the chronological order of a consultation was used. The interviews were pursued until new ideas were exhausted. Patients expressing ideas that were too general or those talking about non-personal experiences were interrupted in order to refocus on a particular personal experience. Each interview lasted 30 minutes on average. All the different wordings of a given idea were recorded. Interviews were transcribed and items were generated from the verbatim statements (n = 105 items).

A literature review was carried out on validated satisfaction questionnaires [5,13-20,23-26,28-30]. This yielded a preliminary list of areas of satisfaction with consultation. Items found in the literature but not in the interviews were collated (n = 26).

This procedure also identified other factors related to outpatient satisfaction with consultation (patient and physician profiles), relevant for inclusion in the questionnaire. The aim was to select the variables linked to satisfaction, independent from place of consultation (department), for the final questionnaire. These variables constitute background adjustment factors needed to avoid bias in comparing departments one with another or the same department over time (age of the patient for example) [31].

A list of satisfaction items (n = 131) was constructed classified into the following domains: administrative procedures, appointment making, receptionist and nurses, waiting time, facilities, duration and privacy of the consultation with the doctor, human relationships with the doctor, information provided by the doctor and shared decision-making, doctor's technical competence, coordination and continuity of care, and global satisfaction. The source of items (interview v. literature) was indicated.

Using the Delphi technique [32], the steering committee and six patients (members of the National League against Cancer) selected items within each domain (n = 60). The number of items to be chosen was proportional to the number of items proposed in each domain. The list of items was submitted as often as necessary to obtain a consensus of at least 80% among the raters.

A focus group (one two-hour meeting) coordinated by two of the authors (IG, SV) including two patient association representatives and three patients, with previous individual access to the list of items, checked acceptability of item wording and exhaustiveness of the list.

A pilot study was conducted on 55 outpatients from different outpatient departments using a preliminary questionnaire comprising the selected items, to check comprehensibility and acceptability of items and response patterns. Confusing items were removed, rewritten or replaced. The list of the items extracted from this qualitative phase is shown on the appendix.

### Questionnaire

The questionnaire obtained from the qualitative phase and tested in the first study comprised 41 negatively and positively worded satisfaction items (Appendix [see Additional File 1]). The traditional approach was chosen, in which the item is structured as a statement of opinion. A Likert five-point response balanced scale was chosen (in French : 'yes certainly', 'yes probably', 'neither yes nor no', 'probably not, 'certainly not') because it seems to be the best format [5,33] and the most often used [5,13,14,17-19,24,28,29]. A 'does not apply' category was provided for 19 items relating to situations not universally relevant. Each item was scored from 0 to 4, 4 indicating greatest of satisfaction. Non-response and 'does not apply' categories were considered as missing data. Patients were asked to answer for their last consultation in the department.

Several other items on general satisfaction were also included in this questionnaire: one overall satisfaction item, using a seven-point scale (from 'not at all' to 'completely' satisfied) and two items on intended behavior (to recommend, to consult again), using a four-point scale ('yes certainly', 'yes probably', 'probably not', 'certainly not') and one open-ended question. These items were included to test concurrent validity.

The questionnaire also comprised data on sociodemographic profile, medical status, visit background and characteristics. and an overall satisfaction with life (using a 7 point scale, from 'not at all' to 'completely' satisfied). This last variable was included because of the relationship between affective disposition and the expression of satisfaction [34,35] and because of the relationship between satisfaction with life and satisfaction with care [36].

### Samples and studies design of the quantitative phase

#### First study (first validation phase)

To select items, a first study was conducted in 2001–2002 in 10 wards of 3 short-stay public teaching hospitals of Paris area (Paul Brousse, Bichat and George Pompidou European hospitals). Data was collected in 7 medical departments (internal medicine, rheumatology, 2 cardiology, dermatology, infectious disease, and oncology) and 3 surgical outpatient departments (urology, orthopedic, surgical gynecology).

All consecutive eligible ambulatory patients over 18 years in scheduled consultation with a physician were included, to obtain approximately 100 subjects per department. Patients hospitalized before or immediately after the consultation were excluded. Research assistants approached outpatients immediately after consultation and invited them to participate. Outpatients were randomized prior to being approached. Outpatients randomized in group 1 completed the questionnaire alone immediately after consultation and left it in a box. Patients of group 2 received the questionnaire by mail at home for completion. They were asked to complete and return it by post in a prepaid envelope carrying a neutral address. Non-respondents were sent up to 3 more questionnaires at one-week intervals. To assess reliability over time a sample of 38 respondents from the second group was sent a second questionnaire to return completed, without any reminder.

Finally of the 1548 outpatients approached, 70.9% agreed to participate (n = 1097) and 65.1% completed the questionnaire (n = 1007). Response rates were 57.0% in group 1 and 73.7% in group 2 (40.2% before any reminder, 63 % after one reminder, 69.7% after two and 73.7% after three). Reasons for non-participation were refusal or lack of time (12.9% of the overall sample), language barrier (8.5%), inability for medical reasons (7.2%), other reason (0.6%) and agreement but no return of the questionnaire after 3 reminders (5.8% of the overall sample and 12.0% of group 2). Compared to respondents, the non-respondent group comprised older subjects (60.2% v. 52.6% aged over 50 years, p < 0.001), more foreigners (12.5% v. 29.1%, p < 0.001) and more patients consulting for the first time in the department (28.0% v. 22.4%, p = 0.02). Response rates also differed according to the department (p < 0.001) and the hospital (p < 0.001).

#### Second study (replication phase)

To confirm the results of the previous study, a second study was conducted in the year 2002 in two departments (internal medicine and infectious disease) in one short-stay public teaching hospital. All consecutive outpatients of 18 years and over (not hospitalized immediately after consultation) were included to obtain 100 participants per department. The questionnaires were posted with a prepaid envelope. One reminder was sent 10 days after the first mailing to non-respondents. Participation rate was 33.9% before reminder and 56.5% after (n = 248).

## Results

### First validation phase

#### Item selection

A first selection of items was made from descriptive response distribution for each item. The criteria used to guide item selection/deletion were: high rates of non-response and 'not applicable' response (≥ 20%) except for items where high rates in this response category were expected, ceiling and floor effects (≥ 50%), and unacceptable test-retest reliability (weighted kappa coefficient<0.60). Pragmatic considerations also tempered selection: interest of the item in itself, number of items covering the same domain, redundancy.

Results showed that the proportion of missing responses per item was low. As predictable, for the two items relating to accessibility of the service in case of emergency (items 5 and 6, Appendix [see Additional File 1]) the number of 'does not apply' responses was high (30.7% and 45.0%). A ceiling effect was observed for all items (from 54.4% to 79.6%), except for those on facilities and waiting time (items 10 to 13).

Test-retest reliability was good for 20 items (weighted kappa ≥ 0.7 for 10 items and from 0.6 to 0.69 for 10 items). For 5 items, the coefficient ranged from 0.45 to 0.56. The item on doctors' warnings on side effects of treatment (item 22) had a very low weighted kappa (k = 0.17). At this stage 12 items were discarded. Item 22 was retained for its clinical relevance (Table 1).

**Table 1:** Item description and scaling properties of the questionnaires extracted from the validation phase (26 item version) and from the replication phase

	**Intermediate questionnaire 26-items retained at the and of the first validation phase (first study, n = 1007)**					**Final questionnaire 27-item questionnaire tested at the replication phase (second study, n = 248)**				
	
Title of the scales	Consultation with the doctor	Appointment making	Reception	Waiting time & facilities	Overall scale	Consultation with the doctor	Appoint-ment making	Reception & facilities	Waiting time	Overall scale
	
Items properties										
# of items in the scale	13	6	3	4	26	13	6	5	3	27
# of questionnaires with at least 1/2 of items completed ^(1)^	996	931	1004	1001	1003	235	248	247	244	248
# of items with 'non response' ≥ 20%	0	0	0	0	0	0	0	0	0	0
# of item with 'does not apply' response ≥ 20%	0	2	0	0	2	1	0	0	0	1
# of items with ceiling effect ≥ 50% (≥ 60%)	13 (12)	6 (4)	3 (2)	0 (0)	22 (18)	12 (4)	1 (1)	2 (0)	0 (0)	15 (5)
# of items floor effect ≥ 50%	0	0	0	0		0	0	0	0	0
range of Weighted kappa (# of items with kappa ≥ 0.60)	0.14–0.83 (10)	0.46–0.77 (3)	0.45–0.78 (1)	0.68–0.82 (4)	0.14–0.82 (18)	-	-	-	-	
Scaling properties										
Mean score (± sd)	85.1 (17.2)	83.2 (19.9)	88.0 (14.5)	69.6 (24.9)	82.7 (13.7)	84.1 (17.2)	80.6 (18.5)	75.3 (18.3)	61.3 (19.6)	78.9 (15.3)
Ceiling / floor effect (%)	26.2 / 0.10	32.2 / 0.2	38.5 / 0.1	16.2 / 1.1	4.2 / 0.1	25.8 / 0.4	24.7 / 0.4	13.7 / 0.5	19.0 / 4.9	4.4 / 0.4
Skewness value /SE	-3.00	-2.09	-3.5	-0.86	-2.67	-0.98	-0.83	-0.58	-0.20	-0.76
Range of interscale correlations	0.33–0.35	0.34–0.37	0.35–0.40	0.33–0.40	-	0.46–0.51	0.51–0.59	0.42–0.49	0.42–0.53	-
# of items with own scale correlation ≥ 0.40 ^(3)^	12	6	2	4	-	13	6	5	3	-
# of items with own scale ^(3) ^correlation greater than with other scale	13	6	3	4	-	13	6	4	3	-
Cronbach alpha coefficient	0.85	0.82	0.69	0.77	0.88	0.94	0.87	0.79	0.89	0.94
Intraclass coefficient [IC95%]	0.69 [0.49–0.83]	0.84 [0.71–0.91]	0.86 [0.75–0.92]	0.83 [0.71–0.91]	0.90 [0.81–0.94]	-	-	-	-	

(1) Including non response and 'does not apply' response' (2) From the final principal component factor analysis (3) Corrected for overlap

#### Factorial structure

The 29 items retained were entered into principal-components factor analysis (PCFA) with 'varimax' rotation and the 26 items with substantial loading ≥ 0.40) on only one factor were retained (Appendix [see Additional File 1]). Another PCFA was computed on the 26 remaining items to determine the structure of the instrument. The screeplot revealed a predominant eigenvalue with nevertheless a four-dimensional structure (the following eigenvalues showed a smooth decrease). Hence the proposal is to consider a four-dimension structure with the possibility of an overall score. One dimension grouped the 13 items relating to consultation with the physician. The second dimension grouped the 6 variables relating to appointment-making. The third and fourth related respectively to waiting time or facilities (4 items) and reception (3 items).

None of the 26 items loaded on more than one factor. Only item 26 ('doctor in touch with attending physician') had a borderline loading (0.37), but it was kept because coordination of care in hospital care is important.

The stability of the 4 factors was ascertained with PCFA on subgroups and with 'oblique' rotation (male v. female and surgery v. medicine).

#### Scale properties

Scores for each scale were based on the standardized sum of the items, giving a range from 0 (low satisfaction) to 100 (high satisfaction). Scores were computed when at least half the items in a scale were completed. Because of a ceiling effect, mean scale scores are relatively high except for the 'waiting time and facilities' scale (Table 1).

Interscale correlations were good for the four scales. One item had a borderline correlation with its own scale (r = 0.37 for item 7 'the consultation room was clearly sign-posted') and one item had a low correlation (r = 0.33 for item 26 'doctor in touch with attending physician'). All items had a higher correlation to their hypothesized scales than to other scales.

Reliability was good, meeting both Cronbach alpha and intraclass correlation coefficient requirements (Table 1).

A very strong association between the overall scale, intended behaviors, comments and global satisfaction question was noted, suggesting good concurrent validity (Table 2).

**Table 2:** Association between overall satisfaction scale, intended behaviors and global satisfaction item from the first validation study (n = 1007) and replication study (n = 248)

	**First validation phase First study (n = 1007)**			**Replication phase Second study (n = 248)**		
		
	n	Score (sd) ^(1)^	P	n	Score (sd) ^(2)^	P
Overall satisfaction item ^(3)^						
1 (*not at all satisfied*)	13	58.0 (21.6)				
2	12	68.7 (17.7)				
3	18	56.0 (13.5)	<0.001 ^(4)^	-	na	-
4	39	59.4 (14.0)				
5	125	72.1 (11.5)				
6	285	80.3 (10.0)				
7 (*completely satisfied*)	496	90.6 (7.6)				
To recommend to relatives or friends						
certainly not	13	52.7 (18.2)	<0.001 ^(4)^	-	na	-
probably not	30	57.3 (16.7)				
yes probably	272	75.4 (13.1)				
yes certainly	665	87.6 (9.5)				
To consult again						
certainly not	10	58.7 (24.2)	<0.001 ^(4)^	-	na	-
probably not	20	57.3 (17.9)				
yes probably	213	72.5 (14.5)				
yes certainly	756	86.6 (10.3)				
To consult again				9		
Do not agree	-	na	-	46	57.8 (14.4)	<0.001
agree				18	64.2 (12.4)	
Fully agree				1	84.3 (11.7	
Content of the open-ended question						
negative comment	303	76.1 (14.7)		85	72.1 (14.9)	
mixed comment	110	78.4 (13.5)	<0.001	8	81.0 (12.4)	<0.001
no comment	442	85.4 (12.1)		91	82.0 (15.8)	
positive comment	152	91.2 (8.2)		42	85.5 (11.1)	

na: non available (1) Overall satisfaction score (26 items scale extracted from the first study) (2) Overall satisfaction score (27 items final scale extracted from the second study) (3) 7-point scale from 1 'not at all satisfied' to 7 'completely satisfied' (4) ANOVA test regrouping the responses 1,2 and 3

### Replication phase

#### Questionnaire tested (see appendix)

A modified version of the questionnaire was constructed at the end of the previous step. To avoid the ceiling effect highlighted in the previous stage, responses choices were modified (using the pattern 'fully agree', 'agree', 'moderately agree, 'not really agree', 'not agree at all'). One satisfaction item on waiting time was added and one item on the facility was reworded to improve the chance of revealing a 'waiting time' subscale and a 'reception-facilities' subscale) and because reliability of the 'reception' subscale was borderline. Patient demographic variables identified at the previous stage as having a relationship with satisfaction scores, one item on intended behavior and an open-ended comment field were also added to the questionnaire.

#### Final psychometric properties of the final 27-item version questionnaire

The number of items with ceiling effect decreased. Item completion rates were good (Table 1). PCFA was performed on the 27 items. The screeplot highlighted the same internal structure. The 'varimax' rotation revealed that two dimensions were identical to those identified in the first study ('consultation with the doctor' and 'contact-appointment') (Table 4). The two others were slightly altered: the three items on 'waiting time' were isolated from items about 'facilities' that grouped themselves with the 'reception' factor. All items had a good loading on their own factor. Item 9 ('pleasantness and availability of receptionist') was the only item with secondary loading on another component. It was kept because it was the only item on human qualities of non medical staff which were cited very often by patients in the qualitative phase (Table 1).

**Table 4:** Principal components factor analysis (varimax rotation) computed with the final 27-items version of the questionnaire (second study, n = 248)

		**Factor 1**	**Factor 2**	**Factor 3**	**Factor 4**
		
		**Consultation with the doctor**	**Appointment making**	**Reception & facilities**	**Waiting time**
1	easy to make an appointment by phone	0.07	**0.49**	0.12	0.39
2	Pleasantness of staff answering the phone	0.24	**0.56**	0.30	0.19
3	Acceptable time lapse to obtain appointment	0.19	**0.81**	0.22	0.18
4	Possibility of obtaining an appointment on convenient day and hour	0.20	**0.77**	0.20	0.14
5	Contacting someone in the facility on the phone for help or advice in case of problem	0.30	**0.68**	0.18	0.08
6	In an emergency, getting a quick appointment in the facility	0.19	**0.77**	0.24	0.11
7	Inside the hospital the consultation room was clearly sign-posted'	0.17	0.15	**0.67**	- 0.16
8	Administrative procedures (completing papers and paying) fast and easy	0.15	0.25	**0.61**	0.16
9	Pleasantness and availability of receptionist	0.20	***0.44***	**0.59**	0.23
10	Waiting room pleasant	0.11	0.15	**0.78**	0.26
11	Premises clean	0.17	0.22	**0.66**	0.18
12	Saw the doctor at the appointed time	0.24	0.18	0.14	**0.84**
13	Waiting time acceptable	0.25	0.29	0.10	**0.85**
14	Information on how long to plan for	0.23	0.16	0.20	**0.78**
15	The doctor was welcoming	**0.69**	0.09	0.38	0.08
16	Took an interest in me not just my medical problem	**0.72**	0.06	0.15	0.18
17	spent adequate time with me	**0.82**	0.14	0.21	0.07
18	Examined me carefully	**0.78**	0.04	0.21	0.07
19	Explained what he/she was doing during the consultation	**0.78**	0.15	0.17	0.12
20	Wanted to know if I had pain	**0.75**	0.13	0.17	0.15
21	Asked if I was taking medication for other health problems	**0.72**	0.07	0.08	0.09
22	Warned me about possible side effects of treatment (operation, drugs)-	**0.68**	0.30	0.01	0.19
23	Took my opinion into account	**0.79**	0.20	0.02	0.10
24	Explained decisions	**0.76**	0.25	0.18	0.20
25	I got the information I wanted	**0.78**	0.23	0.12	0.17
26	he/she is in touch with my GP	**0.57**	0.20	0.02	0.15
27	Agree with doctor's instruction	**0.49**	0.32	0.04	- 0.06

For item-scale correlations, item 9 also correlated to these two scales ('reception-facilities' factor and 'contact-appointment'). It was decided to attribute it to the factor that maximized internal consistency ('reception-facilities' scale). All items met the requirement of being highly correlated to their own scale, all interscale correlations were satisfying, as well as internal consistency (Table 1). The scale overall was significantly associated with comments and intended behaviors (Table 2).

### Construction of a unidimensional 9-item form

As the factorial analysis of both the first validation and replication phases revealed a predominant factor that could be split into four underlying dimensions, it was decided to construct a unidimensional form of the questionnaire, that could produce an overall global outcome that could be very useful in case of evaluation study. Within each dimension, one third of the items were selected according to two criteria: items without 'not applicable' response choice, and items having strong loading on the principal component in PCFA. Thus 9 items were selected, 4 items from the 'consultation with the doctor' scale, 2 from the 'contact-appointment' scale, 2 from 'reception-facilities' and 1 from 'waiting time' (Appendix [see Additional File 1]).

Final PCFA on these 9 items showed scale unidimensionality. Item loading on this factor ranged from 0.56 to 0.78. Item-scale correlation corrected for overlap ranged from 0.47 to 0.65. Internal consistency was good (Cronbach α = 0.86).

### Effect of mode of questionnaire administration on estimation of patient satisfaction

**Figure 1:**
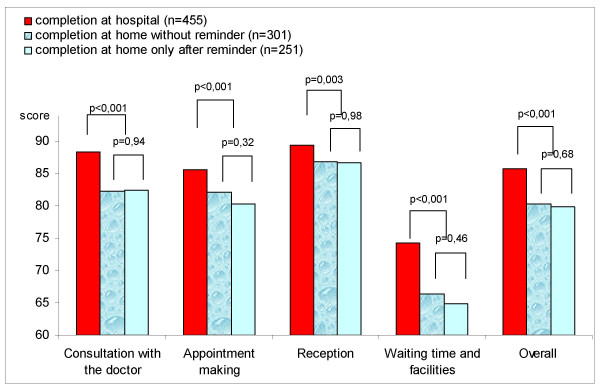
Satisfaction scores according to the place of completion and time of answering (before v. after reminder) (first study, n = 1007).

First study showed that compared to the satisfaction score obtained with completion at home, mean scores for all hospital-completed satisfaction scales were very significantly higher. In the group that completed the questionnaire at home, comparison between respondents before any reminder and respondents after reminder(s) showed no difference in satisfaction scores, whatever the scale considered (Figure 1 – Satisfaction scores according to the place of completion and time of answering [before v. after reminder]).

### Differences between departments

A multiple linear regression showed that differences between departments were highly significant, even if patient characteristics that influenced patients' satisfaction were taken into account (i.e. age, satisfaction with life and previous consultation). Satisfaction scores ranged from 79.3 to 91.7 for 'consultation with the doctor' scale, from 72.8 to 94.2 for 'appointment making' scale, from 83.4 to 91.3 for 'reception' scale, from 57.3 to 80.5 for 'waiting time-facilities' and from 77.8 to 89.3 for the overall scale).

Older age, good satisfaction with life and numerous previous consultations in the department were all associated with high levels of satisfaction, independently from the department (Table 3).

**Table 3:** Association between demographic, medical, outpatient consultation characteristics considered as explanatory variables and overall satisfaction score as dependant variable (1) (linear regression analysis from the first study)

	**DF**	**F-value**	**P-value**
**Demographic profile**			
Age (quantitative variable)	1	7.75	**0.006**
Matrimonial status (Married or living with partner v. single v. divorced, separated, or widowed)	2	0.93	0.42
Working status (employed v. student v. unemployed v. Retired v. prolonged sick leave v. other)	5	0.79	0.56
Level of education (university yes v. no)	1	1.72	0.19
**Overall satisfaction with life **(quantitative variable)	1	51.1	**0.001**
**Modes of care provision**			
Outpatient department (n = 10)	9	4.3	**0.001**
# of consultations in the department (first v. 2 to 3 v. 4 to 5 v. more then 5)	3	2.92	**0.03**
At least one hospitalization in the ward	1	0.73	0.39
**Medical profile**			
Duration of the health problem justifying the consultation (less than 6 month v. 6 month and more)	1	FGF	0.22
Severe medical problem ('yes definitely' v. 'yes rather' v. 'neither yes nor no', v. 'not really', v. 'definitely not' v. 'do not know')	5	1.19	0.31
Comorbidity (yes v. no)	1	1.97	0.16
Perceived health status, compared to persons of same age (better v. similar v. worse)	2	0.49	0.61

829 observations used in the analysis, r^2 ^= 0.21 (1) First short version of the satisfaction questionnaire List of other variables not entered into the model because of non significance in the bivariate analysis (p > 0.1): gender, nationality, gender of the physician, motive of the consultation (physical, psychological or mixed) and prescription of test or medication and having a general practitioner.

## Discussion

### Psychometric properties of the scale

The 27-item and 9-item versions of the questionnaire developed here appear sufficiently concise, valid and reliable to provide a non-biased subjective evaluation of outpatient viewpoint on the quality of care and services in hospital consultations. The questionnaire demonstrated very good internal consistency and good reliability over time. The construction strategy presented here follows most of the recommendations for "good practice" in validation of measurement tools of patient satisfaction with care [7]. Questionnaire content comprises culture-specific features, but overall remains consistent with various north American and European studies [21,23,26,37].

The predominant role given to patients in the early development stages, the literature review and the implication of various experts ensure good content, construct and face validity. This first qualitative step, often insufficiently detailed and structured in satisfaction questionnaire construction, is indeed crucial [38].

The quantitative phase (i.e. first validation with replication) used not only statistical and psychometric results to reach decisions, but also the "intrinsic" and "clinical" relevance of items. This is a very important point. First, because satisfaction studies aim not only to measure quality from user viewpoint, but also to highlight practical elements that can be modified to improve quality. Second, questionnaires that are perceived to have content validity are needed to generate interest in results among health professionals and provide incentive for changes in approach to their jobs. Third, the tendency of health professionals to develop "home-made" questionnaires and their reluctance to use validated questionnaires developed elsewhere can be countered if questionnaire items are perceived as relevant.

### Dimensions of the questionnaire

Each dimension comprises items exploring both technical aspects of care (i.e. equipment, competence, accessibility, continuity, compliance, pain management, waiting and consultation time...) and interpersonal aspects of care (i.e. information, decision sharing, attitude...). These aspects are both predictors of patient opinion on care and services [22,23,37] because implementation of appropriate technical medical strategies is necessary, but not sufficient, to achieve desired outcome. Good management of the human is needed because, as Donabedian remarks, "the interpersonal process is the vehicle by which technical care is implemented and on which its success depends" [1]. According to this author, technical and interpersonal performances are the first circle around the "bull's eye" of the "quality of care" target.

The most important dimension explaining outpatient opinion of hospital quality is the actual consultation with the physician, representing half the items in the tool. This is consistent with other generic patient questionnaires on satisfaction with ambulatory care, also comprising a majority of items related to the medical intervention [17,19,21,26,29,37].

No independent subscales regarding specific aspects of the patient-physician encounter (i.e. communication, professional competence, interpersonal skills...) were identified here. They have been regularly identified by authors developing GP satisfaction questionnaires [5,17,29,37,39,40]. This could be explained by the fact that, as hypothesized, expectations of outpatients with respect to hospital care differs from expectations from primary care. Possibly patients have different needs and expectations according to the type of consultation, hospital specialists generating more mixed expectations because the specific technical competence of hospital specialists predominates and patients have greater difficulty in dichotomizing doctors' skills into "affective " and "technical' dimensions [23,41,42], whereas "affective" qualities have a predominant role in primary care [15,16,43,44]. This is corroborated by the fact that generic questionnaires designed to evaluate hospital care (inpatient or outpatient) most often do not identify such human versus technical dimensions [8,10,26,45,46].

The three other dimensions ('contact-appointments', 'reception-facilities' and 'waiting time') are all related to organizational non-medical aspects of care. These dimensions are classically identified in other generic questionnaires [17,18,21,23,28,29,40,42]. Comparison of the two factorial structures shows stability for all dimensions except 'reception-facilities' and 'waiting time'. From a strictly psychometric viewpoint, these two dimensions, both exploring events occurring just care quality, these two dimensions can pinpoint independent improvement measures, and calculating two different scores may improve the probability of highlighting the impact of such measures.

### Differences between departments and role of background factors

It was shown that satisfaction scores were strongly related to consultation department, regardless of outpatient, physician and care-provision characteristics. These results suggest that this measure is more sensitive to levels of department performance than to patient profile or to modes of consultation, as shown elsewhere [47]. Therefore it is important that each department should identify its weak points to implement specific targeted actions to improve care quality.

As in numerous studies, it was observed that older patients have a higher opinion of care provided than others [7,23,48]. For several authors, this contributes to construct validity of satisfaction questionnaires [41].

The same was observed for patients with multiple contacts with a department [10]. This could be explained by a better match between expectations and experience for multiple consultants, dissatisfaction during first contact leading patients to consult elsewhere.

The strong relationship between overall satisfaction with life and opinion on care expresses the influence of the individual affective disposition trait (i.e. general tendency of an individual to be optimistic or pessimistic) which influences job satisfaction, a concept very close to patient satisfaction with care [49]. Other studies have found relationships between satisfaction and variables strongly associated with perception of overall quality of life, like mental health status and health-related quality of life [12,24].

The influence of these three background factors suggests the need to adjust patient satisfaction scores on these three variables (i.e. patient age, number of contacts and satisfaction with life) when comparing performances between departments or measuring performance over time within departments [31].

### Impact of data collection method

For patients completing the questionnaire immediately after consultation in the hospital, satisfaction estimates were higher than in case of home completion, in spite of procedures to preserve anonymity and confidentiality at hospital. Little data exists on the impact of place of completion for self-administered questionnaires on satisfaction with consultation: two studies conclude that patients express less satisfaction when the questionnaire is completed at home rather than in the medical facility [13,50] and one concluded that there was no difference according to data collection methods, but lacked power because of small sample size [51]. This could be interpreted as an over-estimation of patient satisfaction in case of completion in the facility, patients being more prone to express their real opinion when they have more time to consider the consultation and are safely back home [13]. Moreover response rates in the hospital completion group were relatively low (57%) expressing both refusal to participate or inability to respond, and reluctance to answer a satisfaction questionnaire immediately after consultation because of long waiting time beforehand, or because a relative, an ambulance or a taxi is waiting to take the patient home. It could be concluded that completion at home may be better than immediately after consultation.

In the present study, no difference was observed between respondents without reminder and respondents only after reminder(s). This result is in agreement with other studies assessing inpatient satisfaction [9,52]. It could be concluded that reminders are not necessary to produce non-biased data.

## Limitations

This work entails several limitations. First, overall response rates only reached 65% despite reminders sent to patients receiving mailed questionnaires at home. However, unlike other studies, the response rates calculated did not exclude patients unable to respond for medical reason (i.e. who were very ill or did not understand French) and homeless patients giving an invalid address (shelter...).

Second, non-respondents differed from respondents regarding two background factors influencing satisfaction levels, with over-sampling of less-satisfied subjects in the respondent group (young patients and first consultants). There are also differences in participation rates between departments that could lead to over-estimating real differences between departments, because the more satisfied outpatients within each department may have been excluded.

Third, validation is a continuous process and further studies are required to confirm these first results. The experimental nature of these studies may have induced bias in questionnaire responses. So there is a need to replicate findings using confirmatory statistical methods (IRT or structural equation model for example) using the data from non experimental, routine studies.

## Conclusion

Good estimation of patient opinion on hospital consultation can be obtained with these two questionnaires. When comparing performances between departments or the same department over time scores need to be adjusted on the three variables that influence satisfaction independently from department (patient age, previous consultation in the department and overall satisfaction with life score). Mail-back completion at home of the questionnaire seemed preferable to completion in the consultation facility immediately after the consultation. Reminders are not necessary to produce non-biased data.

## Authors' contribution

IG – initiation of the research, supervision of the project and drafting the manuscript; SV – coordination of the 2 studies, participation in the interpretation of the results and revision of the draft paper; CDS – performing statistical analyses; PD and PR- participation in the conception, design and coordination of the research; BF – participation in the interpretation of the results, supervision of the statistical analysis and revision of the draft paper.
